# SMOKING AND SECONDHAND SMOKE: Study Finds No Level of SHS Exposure Free of Effects

**DOI:** 10.1289/ehp.118-a474a

**Published:** 2010-11

**Authors:** Carol Potera

**Affiliations:** **Carol Potera**, based in Montana, has written for *EHP* since 1996. She also writes for *Microbe*, *Genetic Engineering News*, and the *American Journal of Nursing*

How much exposure to tobacco smoke can the lungs endure before damage ensues? The answer appears to be none, based on gene activity measured by researchers at Cornell University.^1^ “No level of smoking or exposure to secondhand smoke [SHS] is safe. Even at the lowest detectable levels of exposure, we could detect changes in gene expression within the cells lining the airways,” says coauthor Ronald Crystal, head of pulmonary and critical care medicine at New York-Presbyterian/Weill Cornell Medical Center.

Crystal and coworkers at Cornell analyzed gene activity in small airway epithelial cells collected from 121 healthy volunteers. The type of cells tested are where early damage first occurs that leads to chronic obstructive pulmonary disease (COPD) and bronchogenic cancer, according to Crystal.

The volunteers, all of whom had normal lung function, were categorized by tobacco smoke exposure status as determined by their urine levels of nicotine and cotinine. Nonsmokers had nondetectable urine nicotine or cotinine levels, low-exposure individuals had urine nicotine and/or cotinine levels up to 1,000 ng/mL, and active smokers had urine nicotine and/or cotinine levels greater than 1,000 ng/mL. The low-exposure group included occasional smokers and people exposed to SHS.

The researchers first compared the smokers and nonsmokers. Microarrays detected significant changes between these two groups in the activity of 372 genes. Among the low-exposure group, about a third of these 372 genes were up- or downregulated compared with nonsmokers, and 11% of the genes differed compared with active smokers.^1^

Even subjects with the lowest levels of nicotine and cotinine had enhanced activity of biological pathways involved in the metabolism of xenobiotics by cytochrome P450 and arachidonic acid. The same two pathways also were highly activated in smokers, suggesting exposure to low levels of SHS caused changes in the airways similar to those from active smoking, representing the earliest biologic abnormalities that can lead to disease.^1^ The authors believe this may be the first study to document biological changes in the lung cells of people exposed to low levels of tobacco smoke.

The results support epidemiologic studies that link early respiratory damage to low levels of SHS exposure or occasional smoking.^2^,^3^ However, the tobacco smoke–induced gene changes “do not tell us which ones [genes] are dangerous and which are protective,” Crystal notes.

Moreover, the cross-sectional nature of the study precluded determining whether the genetic changes predicted disease. Followup studies lasting 20 years or more are needed to sort out the genes that play a role in the development of lung diseases, and Crystal plans to follow some of the people in this study.

People often wonder what level of exposure to SHS is harmful—is it a problem, for instance, to hang out with smoking friends once or twice a week? Crystal’s study “employs sophisticated molecular genetic techniques to address this very important public health question of whether a threshold exists,” says Norman Edelman, a professor of preventive medicine at Stony Brook University Medical Center and chief medical officer at the American Lung Association. The finding that no level of tobacco smoke exposure appeared safe “is important for informing both individual behavior and public health policy,” Edelman says.
